# Quantitative Proteomic Analysis of ER Stress Response Reveals both Common and Specific Features in Two Contrasting Ecotypes of *Arabidopsis thaliana*

**DOI:** 10.3390/ijms21249741

**Published:** 2020-12-21

**Authors:** Yu-Shu Lyu, Yu-Jian Shao, Zheng-Ting Yang, Jian-Xiang Liu

**Affiliations:** 1 State Key Laboratory of Plant Physiology and Biochemistry, College of Life Sciences, Zhejiang University, Hangzhou 310027, China; luyushu90@163.com (Y.-S.L.); yjshao@zju.edu.cn (Y.-J.S.); 2 School of Life Sciences, Guizhou Normal University, Guiyang 550018, China; zhengtingyang@gznu.edu.cn

**Keywords:** *Arabidopsis thaliana*, ecotype, ER stress, proteomics, TMT, UPR

## Abstract

Accumulation of unfolded and misfolded proteins in endoplasmic reticulum (ER) elicits a well-conserved response called the unfolded protein response (UPR), which triggers the upregulation of downstream genes involved in protein folding, vesicle trafficking, and ER-associated degradation (ERAD). Although dynamic transcriptomic responses and the underlying major transcriptional regulators in ER stress response in Arabidopsis have been well established, the proteome changes induced by ER stress have not been reported in Arabidopsis. In the current study, we found that the Arabidopsis Landsberg erecta (L*er*) ecotype was more sensitive to ER stress than the Columbia (Col) ecotype. Quantitative mass spectrometry analysis with Tandem Mass Tag (TMT) isobaric labeling showed that, in total, 7439 and 7035 proteins were identified from Col and L*er* seedlings, with 88 and 113 differentially regulated (FC > 1.3 or <0.7, *p* < 0.05) proteins by ER stress in Col and L*er*, respectively. Among them, 40 proteins were commonly upregulated in Col and L*er*, among which 10 were not upregulated in *bzip28 bzip60* double mutant (Col background) plants. Of the 19 specifically upregulated proteins in Col, as compared with that in L*er*, components in ERAD, N-glycosylation, vesicle trafficking, and molecular chaperones were represented. Quantitative RT-PCR showed that transcripts of eight out of 19 proteins were not upregulated (FC > 1.3 or <0.7, *p* < 0.05) by ER stress in Col ecotype, while transcripts of 11 out of 19 proteins were upregulated by ER stress in both ecotypes with no obvious differences in fold change between Col and L*er*. Our results experimentally demonstrated the robust ER stress response at the proteome level in plants and revealed differentially regulated proteins that may contribute to the differed ER stress sensitivity between Col and L*er* ecotypes in Arabidopsis.

## 1. Introduction

In eukaryotic cells, protein folding machineries in the secretory pathways are well maintained to ensure the accuracy of protein folding and timely elimination of misfolded proteins accumulated in the endoplasmic reticulum (ER) [1]. When unfolded and misfolded proteins are built-up in ER during plant development and its adaptation to environmental stresses, a well-conserved response called the unfolded protein response (UPR), is elicited to sense transduce signals in the ER and regulate gene expression in the nucleus, enhancing the protein folding capacity of ER to cope with the protein folding demands [2]. 

At least four branches of the UPR pathways, namely the regulated intramembrane proteolysis (RIP), regulated IRE1-dependent RNA splicing (RIDS), regulated IRE1-dependent RNA decay (RIDD), and regulated IRE1-dependent protein phosphorylation (RIDP), have been identified in plants that operate to promote cell survival by reducing misfolded protein accumulation in the ER [1]. In the RIP pathway, membrane-associated transcription factors, for example, bZIP28 in *Arabidopsis thaliana*, are activated via protease-mediated processing. Under normal growth conditions, bZIP28 is associated with ER membranes [3,4,5], however, when unfolded or misfolded proteins accumulate in the ER, bZIP28 is dissociated with chaperones (i.e., BiPs), and relocates from the ER to the Golgi, where it is subjected to proteolytic cleavage by site-specific proteases, including the site 2 protease (S2P) [6,7,8]. The N-terminal part of bZIP28, containing DNA-binding and transcriptional activation domains, enters the nucleus and induces the expression of several downstream genes [9,10,11,12]. 

Inositol-requiring enzyme 1 (IRE1) is a key component in the latter three branches, which is an ER membrane-associated protein with a protein kinase domain and an endonuclease domain in the cytosol-facing C-terminus [13]. In Arabidopsis, IRE1A and IRE1B are auto-phosphorylated under ER stress conditions and catalyze the specific splicing of *bZIP60* mRNA within its double stem-loop structure in the RIDS pathway [14,15,16]. The unconventional splicing of *bZIP60* mRNA results in an open reading frame shift and produces a nucleus-localized form of transcription factor bZIP60 [15,16,17]. bZIP28 and bZIP60 are both required for robust transcriptional regulation and ER stress tolerance in Arabidopsis [9,18]. When ER stress becomes severe, IRE1A and IRE1B non-specifically digest mRNAs through a RIDD pathway to reduce the number of proteins entering the ER or promote the induction of autophagy [19,20]. Recently, the endonuclease-independent role of IRE1 has been revealed in Arabidopsis. The protein kinase domain of IRE1 is essential for its role in RIDP to regulate plant growth and development, although the phosphorylation substrates of IRE1 have not yet been identified [21,22,23]. 

In response to persistent ER stress, a multiphasic program of gene expression interwoven among cellular events has been reported in plants [9,24,25,26,27], however, how plant cells respond to ER stress at the proteome level has been explored less. In soybean (*Glycine max* L.) root tips stressed with flooding and drought, gel-free and label-free proteomic analysis has revealed that ER proteins related to protein glycosylation and signaling were differentially accumulated in response to both stresses [28]. Secretion-associated and Ras-related 1 (Sar1), a small GTPase for the assembly of coat protein complex II, exports secretory protein from the ER to the Golgi apparatus. Endosperm-specific knockdown of *OsSar1* in rice (*Oryza sativa* L.) blocked secretory proteins in the ER and elicited an ER stress response in rice seeds [29]. Using an iTRAQ-based proteomics analysis, 405 proteins were found to be differentially regulated in the *OsSar1* RNAi seeds by >2.0-fold as compared with the wild-type control, of which the upregulated proteins were mainly involved in protein modification, transport, and degradation, while the downregulated proteins were mainly involved in metabolism and stress/defense responses [30].

Columbia (Col) and Landsberg erecta (L*er*) are two of the most commonly studied accessions of Arabidopsis ecotypes, with distinct genomic features diverged ∼200,000 years ago [31,32]. These two ecotypes were substantially different from each other in their tolerance to prolonged extreme heat in greenhouse conditions (40 °C), and overexpression of the receptor-like kinase *ERECTA* improved thermotolerance in rice and tomato in greenhouse and field tests at multiple locations [33]. Since ER stress and UPR are associated with heat stress tolerance in plants [26], in the current study, we compare ER stress sensitivity of Col and L*er* plants and found that L*er* is more sensitive to ER stress than Col in the current study. We further applied Tandem Mass Tag (TMT)-based quantitative proteomic analysis of ER stress responses in Col and L*er* and revealed both common and specific features of responses in two contrasting ecotypes. Information from this study provides new insights into ER stress responses at the proteome level in plants and helps in understanding the molecular basis of ER stress tolerance in Col and L*er* ecotypes.

## 2. Results and Discussion

### 2.1. Differed ER Stress Sensitivity in Arabidopsis Ecotypes Columbia (Col) and Landsberg erecta (Ler)

**Figure 1:**
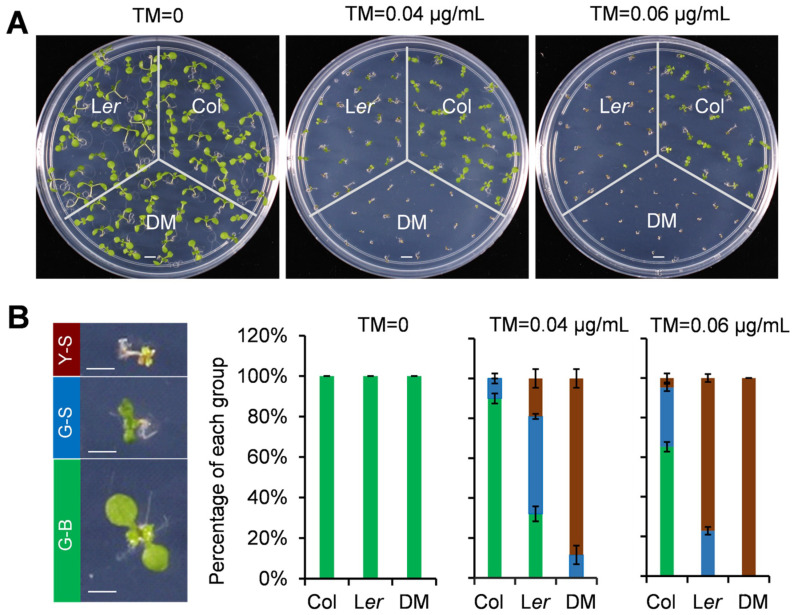
Endoplasmic reticulum (ER) stress sensitivity assays in Columbia (Col), Landsberg erecta (L*er*), and double mutant (DM) plants. (**A**) Wild-type Col and L*er* plants were grown on ½ MS plates without or with various concentrations of tunicamycin (TM) for 9 days, and then photographed; (**B**) The percentage of green-big (G–B), green-small (G-S) and yellow-small (Y-S) plants was calculated after photo-taking. Bars depict SE (*n* = 3). The difference of each group between L*er* and Col under ER stress conditions is significant (*p* < 0.05). The *bzip28 bzip60* double mutant (DM) plants in the Col background was used as a control. Bar = 5 mm.

L*er* is presumably the second-most-used strain of Arabidopsis after the reference accession Columbia, and it is in the accession La-1 background with a mutation in the *ERECTA* gene [32]. Previous studies on UPR in Arabidopsis [2] have usually been carried out in the Col background, however, we were interested in comparing the ER stress sensitivity between Col and L*er*. Under normal growth conditions, no obvious phenotypes were observed among the wild-type of Col, the wild-type of L*er*, and the *bzip28 bzip60* double mutant (DM) plants (Col background) (Figure 1A,B). However, when the growth mediums were supplied with ER stress inducer tunicamycin (TM), similar to the DM plants, the L*er* wild-type plants were more sensitive to TM than the Col wild-type plants (Figure 1A,B). Therefore, the ER stress sensitivity was different between Col and L*er*.

### 2.2. TMT-Based Proteomic Analysis of ER Stress Responses in Col and Ler

**Figure 2:**
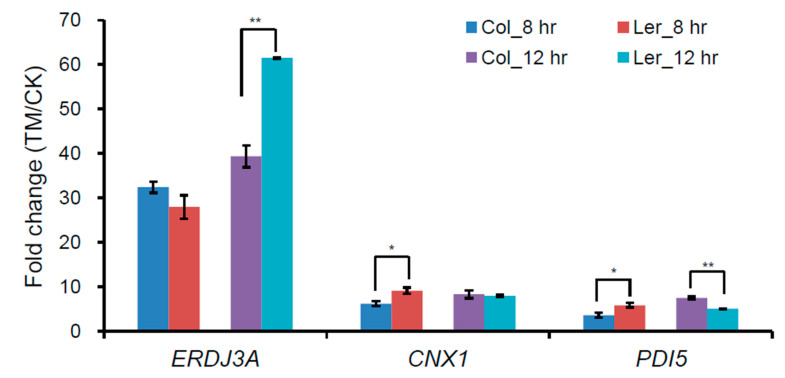
Gene expression analysis of ER stress marker genes in Col and L*er* plants. Nine-day-old Col and L*er* seedlings grown on ½ MS plates were treated without or with 5 μg/mL tunicamycin (TM) for 8 h or 12 h, and total RNA was extracted for quantitative RT-PCR (RT-qPCR). Fold change is the expression level of genes in TM-treated plants relative to that in non-treated (CK) plants, both of which were normalized to that of the control *ACTIN*. Error bars *represent* SE (n = 3). Asterisks indicate significance levels as compared with two ecotypes in *t*-test. (*, *p* < 0.05; **, *p* < 0.01).

**Figure 3:**
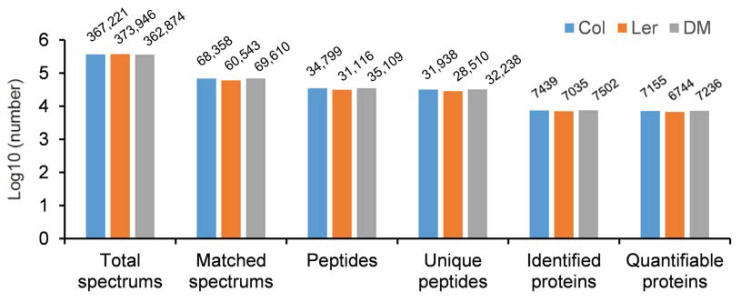
An overview of protein identifications in Col, L*er*, and DM plants. Wild-type Col and L*er* plants were grown on ½ MS plates for 9 days, and then seedlings were treated without or with 5 μg/mL tunicamycin (TM) for 12 h. Total proteins were extracted from the whole seedlings for TMT-based quantitative proteomics analysis. The *bzip28 bzip60* double mutant (DM) plants in the Col background was used as a control.

**Figure 4:**
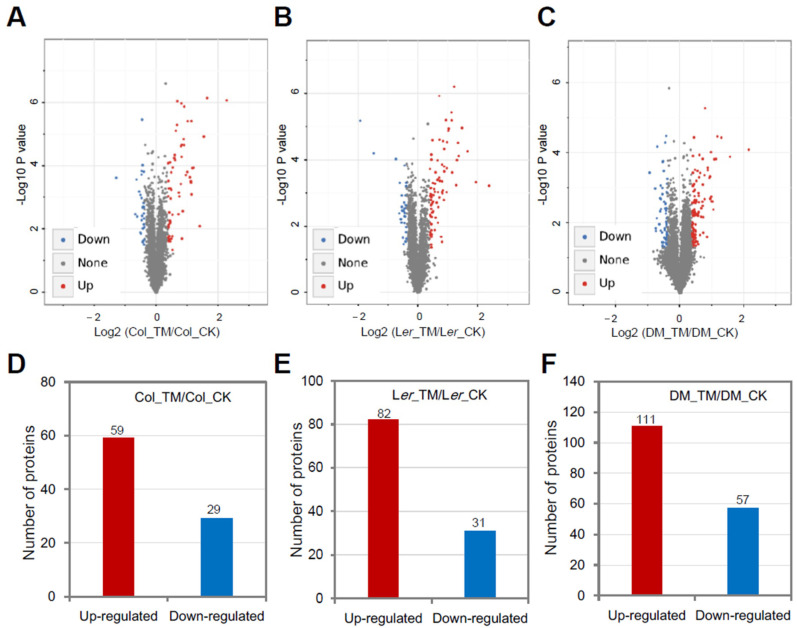
Differentially regulated proteins by ER stress among Col, L*er*, and DM plants. (**A**–**C**) Volcano plots of differential abundance of ER stress-regulated proteins among Col, L*er*, and DM plants. (**D**–**F**) Number of differentially regulated proteins by ER stress in Col, L*er*, or DM plants. Parameters were set as fold change >1.3 (upregulation) or <0.7 (downregulation), *p* < 0.05.

To understand the ER stress sensitive phenotype of L*er*, first, we checked the expression of three UPR downstream marker genes (*ERDJ3A*, *CNX1*, and *PDI5*) in Col and L*er* plants under ER stress conditions, and the results showed that the ER stress-induced upregulation of *ERDJ3A*, *CNX1*, and *PDI5* was quite similar between Col and L*er* at 8 h, the fold induction of *ERDJ3A* was even much higher in L*er* than that in Col (Figure 2), which could not sufficiently explain the observed difference in ER stress sensitivity between these two ecotypes. Although proteomic analysis of ER stress responses has been carried out in human and mouse cells [34,35], there has been no such proteomic study reported in plants. Therefore, we employed TMT-based quantitative proteomics to compare the ER stress responses in Col and L*er* plants. In total, 34,799, 31,024 and 35,109 peptides, corresponding to 7439, 7046, and 7502 proteins, were identified in Col, L*er*, and DM plants, respectively, among which 7155, 6750, and 7236 proteins contained quantifiable information in at least one of the three group of plants (Figure 3). Principal components analysis (PCA) results showed good reproducibility of data for the control (CK) and ER stress treatment (TM) samples in each group (Figure S1). Volcano plot and protein abundance data showed the differential expression of proteins between CK and TM in each group of plants (Figure 4A–C and Table 1). According to the variance analysis with *p* < 0.05 and 1.3- or 0.7-fold change, a commonly used stringent parameter setting for TMT-based comparative proteomics [36,37,38], in total, 59, 82, and 111 quantified proteins were considered to be upregulated, and 29, 31, and 57 proteins were considered to be downregulated proteins at the protein level by ER stress in Col, L*er*, and DM plants, respectively (Figure 4D–F, Table S1). The enrichment of gene ontology (GO) analysis showed that similar biological process (BP) or molecular function (MF) were enriched in Col and L*er*, in which GO terms related to protein folding and degradation were commonly found (Figures S2 and S3). GO terms of cellular component (CC) related to the secretory pathways were enriched in Col, L*er*, and DM plants (Figure S4). These results were consistent with previous transcriptomic analysis that the expression level of molecular chaperones, protein disulfide isomerases, and ER-associated degradation proteins was affected by ER stress in Arabidopsis [9,24,39,40].

### 2.3. Differentially Regulated Proteins by ER Stress between Col and Ler

**Figure 5:**
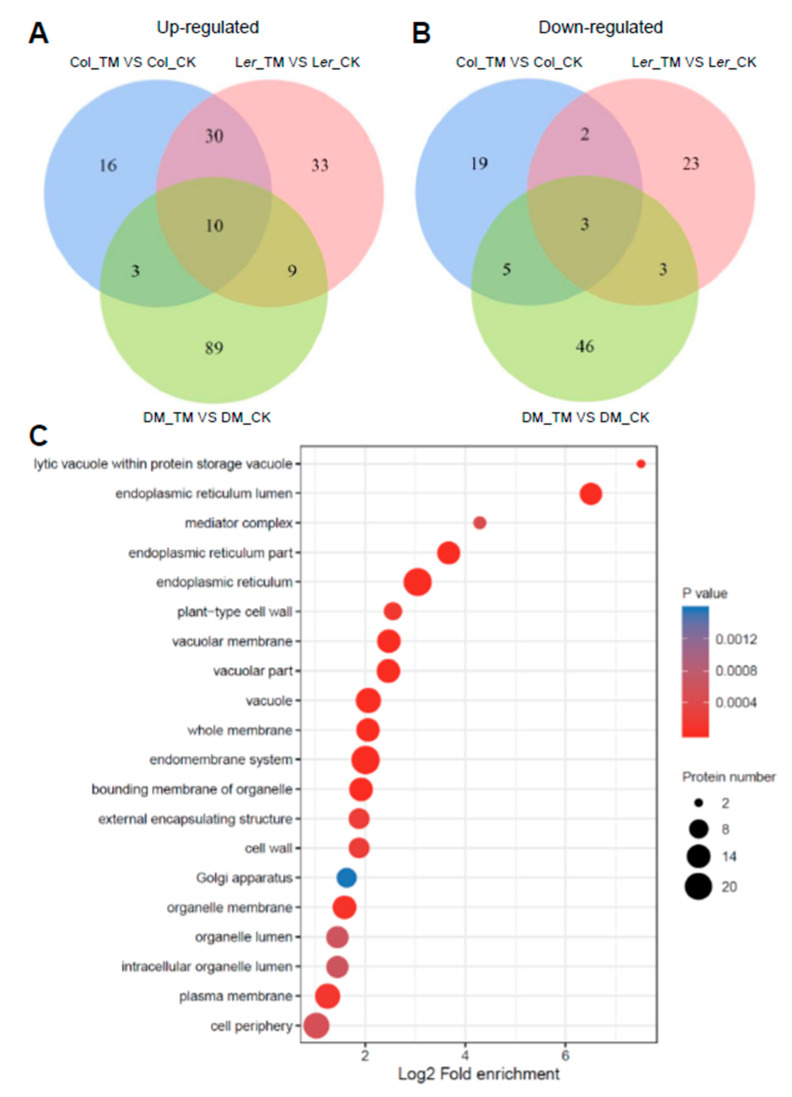
Commonly and specifically regulated proteins at the protein level by ER stress among Col, L*er*, and DM plants. (**A**,**B**) Venn diagrams showing the number of overlapping and non-overlapping proteins that were differentially regulated among Col, L*er*, and DM plants. Criteria for differential regulation were set as fold change >1.3 for upregulation or <0.7 for downregulation, *p* < 0.05; (**C**) Functional enrichment of cellular components for the proteins that are commonly upregulated by ER stress in both Col and L*er* plants.

In order to understand the observed differential ER stress sensitivity between Col and L*er*, we compared the differentially upregulated or downregulated proteins (*p* < 0.05, FC > 1.3, or FC < 0.7) among Col, L*er*, and DM plants. Venn diagrams showed that there were 40 proteins that were commonly upregulated in both Col and L*er*, among which 30 proteins were not upregulated at the protein level in DM plants (Figure 5A and Table S2). These 40 commonly upregulated proteins were enriched in GO terms associated with the secretory subcellular locations (Figure 5C), which was consistent with their role in UPR. There were 19 proteins that were specifically upregulated in Col plants as compared with that in L*er* plants, and 16 of them were not upregulated at the protein level in DM plants (Figure 5A, Table 2 and Table 3, and Table S2). In contrast, there were 24 proteins that were specifically downregulated at the protein level in Col plants as compared with that in L*er* plants (Figure 5B, Table 4 and Table 5, and Table S2), many of which were related to growth and stress responses.

Among the 19 specifically upregulated proteins in Col, five proteins (FLA1, UTR1, UGT71C2, AT3G51000, and AT5G19250) are related to glyco-modification; five proteins (CRT1A, ERO1, FLOT1, SEC62, and AT3G16990) are involved in ER protein folding and trafficking; two proteins (EBS7 and OS9) are important components in ER-associated degradation (ERAD); two proteins (TGA6 and NAC091) are transcription factors (Table 2).

Calnexin (CNX) and calreticulin (CRT) are lectins that recognize oligosaccharide side chains on glycoproteins and form CNX/CRT protein-folding cycles to serve as a quality control system for successful protein folding in ER [41]. Defects in all three Arabidopsis CRT genes affected protein folding and conferred high sensitivity to drought [42]. During CNX/CRT cycles, continuous glucose trimming by glucosidase II (GCSII) and UDP-glucose-dependent re-glucosylation of unfolded glycoproteins by UDP-glucose:glycoprotein glucosyltransferase (UGGT) takes place [43]. Nucleotide sugar transporter is required in the CNX/CRT cycle to transport UDP-glucose from the cytosol to the ER lumen, which is controlled by two proteins UTR1 and UTR3 in Arabidopsis [44,45]. In the CNX/CRT cages, forming and reforming disulfide bonds through repeated oxidation and reduction catalyzed by protein disulfide isomerase (PDI) and other thiodisulfide oxidoreductases are important for correct protein folding in the ER, and the formation of protein disulfide bonds requires oxidizing equivalents, which are supplied by ER oxidoreductin 1 (ERO1) [46,47]. CRT1A, UTR1, and ERO1 are more accumulated at the protein level in Col than that in L*er* under ER stress conditions, suggesting that ER protein folding is more efficient in Col than that in L*er* plants.

Autophagy, another quality control system in plants, selectively targets and degrades damaged organelles (e.g., ER) and misfolded proteins inside the organelles. SEC62, originally considered to be a constituent of the translocon complex regulating protein import in the mammalian ER, also functions as an ER-resident autophagy receptor to selectively deliver ER components to the autolysosomal system for clearance to maintain ER homeostasis [48]. Recent studies have confirmed the role of Arabidopsis SEC62 in ER-phagy under ER stress in which SEC62 likely functioned as an ER-phagy receptor in plants [49,50]. The accumulation of SEC62 at the protein level under ER stress conditions is only detected in Col, which is consistent with the notion that that ER-phagy in Col is more activated than that in L*er* plants.

ERAD is also an ER-mediated protein quality control system that timely recognizes and removes misfolded proteins from the ER to the cytosol for degradation via the 26S proteasome [51]. There are two major complexes of the ERAD system in plants, the HMG-CoA reductase degradation 1 (HRD1) complex and the degradation of alpha2 10 (DOA10) complex [1]. In the conserved HRD1 complex, Arabidopsis osteosarcoma-9 (AtOS9)/EMS bri1 suppressor6 (EBS6), and AtHRD3/EBS5 recognize presumably misfolded ERAD targets [52,53,54], while E2 conjugase UBC32 and E3 ligase HRD1 ubiquitinate and degrade ERAD substrates via the 26S proteasome [53,55]. EBS7 is a plant-specific ERAD component that interacts with and stabilizes HRD1a in the ER membranes, regulating protein stability of the misfolded clients BRI-9 and BRI-5 [56]. In the current proteomics study, the upregulation of EBS7 and OS9 proteins at the protein level was only detected in Col plants, suggesting that the ERAD machineries in Col plants are more efficient than that in L*er* plants, which is correlated to the observed difference in ER stress sensitivity between two ecotypes. 

UPR is an important component of immunity to host pathogens and of systemic acquired resistance (SAR). Mutant plants such as *ire1a*, *ire1b*, and *bzip60* that are compromised in the UPR are more susceptible to bacterial pathogens and less able to establish SAR to the bacteria when treated with salicylic acid (SA) [57]. The bZIP transcription factor TGA6 functions redundantly with TGA2 and TGA5 in SA-mediated SAR [58,59]. The NAC transcription factor NAC091 (also known as TIP) is closely related to NAC062, a plant-specific membrane-associated transcription factor involved in UPR [60]. These two proteins were specifically upregulated at the protein level by ER stress in Col plants. In the future, it is worthwhile to investigate in detail whether TGA6 and/or NAC091 regulate UPR in plants. 

### 2.4. Gene Expression Analysis of Differentially Upregulated Proteins by ER Stress between Col and Ler

**Figure 6:**
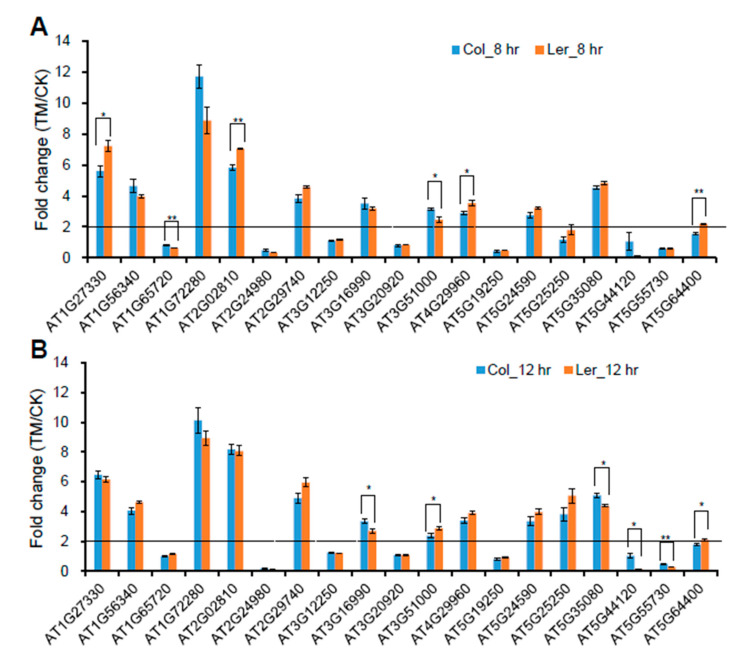
Expression analysis of genes encoding the differentially regulated proteins by ER stress between Col and L*er* plants. (**A**,**B**) Ten-day-old Col and L*er* seedlings grown on ½ MS plates were treated without or with 5 μg/mL tunicamycin (TM) for 8 h (**A**) or 12 h (**B**), and total RNA was extracted for quantitative RT-PCR (RT-qPCR). Fold change is the expression level of genes in TM-treated plants divided by that in control (CK) plants, both of which were normalized to the expression of *ACTIN*. Error bars represent SE (*n* = 3). Asterisks indicate significance levels when comparing two ecotypes in *t*-test. (* *p* < 0.05; ** *p* < 0.01).

In order to know whether the differentially regulated proteins by ER stress between Col and L*er* are correlated to differential gene expression levels, quantitative RT-PCR was performed. The results showed that 11 genes out of 19 genes encoding specifically upregulated proteins in Col plants were upregulated at the transcription level while the remaining eight genes (AT1G65720, AT2G24980, AT3G12250, AT3G20920, AT5G19250, AT5G44120, AT5G55730, and AT5G64400) were not upregulated, even after 12 h of treatment at the transcription level by ER stress treatments (fold change >2, *p* < 0.05) (Figure 6A,B). For the abovementioned 11 genes, none of the denes differed in terms of transcript upregulation ratio (fold change >1.3 or <0.7, *p* < 0.05) in plants between Col and L*er* (Figure 6A,B). The above results indicated that these proteins were regulated by ER stress after gene expression, possibly at translational or post-translation levels in two different Arabidopsis ecotypes. Except for AT5G64400, which was upregulated (fold change >2, *p* < 0.05) in L*er* plants, there were seven out of eight genes, including *SEC62* and *TGA6*, that were not upregulated or downregulated (fold change >2, *p* < 0.05) at the transcription level by ER stress in both ecotypes (Figure 6A,B), suggesting that these encoding proteins were upregulated by ER stress only at the protein level.

## 3. Concluding Remarks

In conclusion, the current study reports the different ER stress sensitivities in two Arabidopsis ecotypes Col and L*er*. Quantitative proteomics reveals the common and specific proteins that are differentially regulated by ER stress at the protein level in these two ecotypes. Among the 19 differentially upregulated proteins between Col and L*er*, transcripts of 11 proteins were upregulated by ER stress at the gene expression level, however, the transcript upregulation ratios of them were not significantly different in plants between Col and L*er*. These 11 proteins are involved in protein folding, vesicle trafficking, and protein degradation, and the relative higher ER stress-induced protein level in Col is correlated to the relative less ER stress sensitivity in Col as comparing with that in L*er*. There are with identified proteins, of which the transcript level is not affected, but the protein level is upregulated by ER stress in Col but not in L*er*, further demonstrating the complementary power of proteomics c in identifying new components in UPR in plants. Future genetic experiments are needed to further dissect the molecular mechanisms underlying elevated ER stress sensitivity in L*er* ecotype in Arabidopsis.

## 4. Materials and Methods

### 4.1. Plant Materials and Endoplasmic Reticulum (ER) Stress Treatment

Wild-type *Arabidopsis thaliana* lines of Col (Col-0) and L*er* (L*er*-0) were used in this study. The *bzip28 bzip60* double mutant (DM) plants were obtained by crossing the T-DNA mutants *bzip28* (SALK_132285) with *bzip60* (SALK_050203), both of which were in the Col background. For ER stress sensitivity assays, seeds were germinated on agar plates containing half-strength Murashige and Skoog (MS) salts, 1.2% sucrose, 0.05% MES, and different concentrations of tunicamycin (TM) (Sigma, CA, USA) as described in the text, pH 5.7. Seedlings were grown in an illuminated growth chamber at 22 °C for 9 days after stratification at 4 °C for 2 days and plant phenotypes were recorded. For proteomics analysis, 9-day-old seedlings grown on 1/2 MS plates were transferred to liquid MS medium plus 5 μg/mL TM for 12 h, and whole seedlings were harvested for protein extraction. For gene expression analysis, 9-day-old seedlings grown on 1/2 MS plates were transferred to liquid MS medium plus 5 μg/mL TM for 8 or 12 h, and whole seedlings were harvested for RNA extraction. There were three biological replicates for both proteomics and gene expression analysis. 

### 4.2. Protein Extraction, Digestion, and TMT Labeling

TMT-based proteomics was done with commercial service available at PTM BIO, Hangzhou, China (http://www.ptm-biolab.com.cn/). Plant tissues were ground in liquid nitrogen and powders were transferred to 5 mL centrifuge tubes. After that, four volumes of lysis buffer (8 M urea, 1% Triton-100, 10 mM dithiothreitol, and 1% Protease Inhibitor Cocktail) was added, followed by sonication three times on ice using a high intensity ultrasonic processor (Scientz, Ningbo, China) and centrifugation at 20,000× *g* at 4 °C for 10 min. Finally, the supernatant was precipitated with cold 20% TCA for 2 h at −20 °C. The protein was dissolved in 8 M urea and the protein concentration was determined with a BCA kit, according to the manufacturer’s instructions. For digestion, the protein solution was reduced (5 mM dithiothreitol, 30 min) at 56 °C and alkylated with iodoacetamide (11 mM, 15 min) at room temperature in the dark. Then, the protein sample was diluted by adding 100 mM triethylamonium bicarbonate (TEAB). Finally, trypsin was added at 1:50 trypsin-to-protein mass ratio for the first digestion overnight and 1:100 trypsin-to-protein mass ratio for a second 4 h digestion. For TMT labeling, peptide was desalted with Strata X C18 SPE column (Phenomenex, CA, USA) and vacuum dried. After that, peptide was reconstituted in 0.5 M TEAB and processed according to the manufacturer’s protocol for the TMT kit. Briefly, one unit of TMT reagent was thawed and reconstituted in acetonitrile. Then, the peptide mixtures were incubated for 2 h at room temperature and pooled, desalted, and dried by vacuum centrifugation.

### 4.3. HPLC Fractionation and LC-MS/MS Analysis

HPLC fractionation was performed with high pH reverse-phase HPLC. Briefly, peptides were dissolved in 0.1% formic acid, and then directly loaded onto reversed-phase analytical columns (15 cm in length, 75 μm i.d.). The gradient was increased from 6% to 23% (0.1% formic acid in 98% acetonitrile) over 26 min, 23% to 35% in 8 min, 35% to 80% in 3 min, and then held at 80% for the last 3 min, all at a constant flow rate (400 nL/min) on an EASY-nLC 1000 UPLC system. The peptides were sent to NSI source followed by tandem mass spectrometry (MS/MS) in Q ExactiveTM Plus (Thermo, CA, USA) coupled online to the UPLC in the Orbitrap with routine setting parameters. The mass spectrometry proteomics data were deposited to the ProteomeXchange Consortium via the PRIDE partner repository with the dataset identifier PXD022630.

### 4.4. Database Search and Bioinformatics Analysis

The resulting MS/MS data were processed using Maxquant search engine (v.1.5.2.8). Tandem mass spectra were searched against the Uniprot database concatenated with reverse decoy database using Col or L*er* sequences. Trypsin/P was chosen as the cleavage enzyme allowing up to 2 missing cleavages. The mass tolerance for precursor ions was set as 10 ppm in First search and 5 ppm in Main search, and the mass tolerance for fragment ions was set as 0.02 Da. Cys carbamidomethylation was specified as a fixed modification, and acetylation modification and oxidation on Met were specified as variable modifications. FDR was adjusted to <1% and minimum score for modified peptides was set to >40. Differential expression proteins were obtained based on fold changes and P values as indicated in the text. Gene ontology (GO) annotations were retrieved from the UniProt-GOA database (http://www.ebi.ac.uk/GOA/) and classified as three categories, i.e., biological process, cellular component and molecular function. For each category, a two-tailed Fisher’s exact test was employed to test the enrichment of the differentially expressed protein against all identified proteins. The GO with a corrected p-value <0.05 is considered significant.

### 4.5. Quantitative Gene Expression Analysis

RNA from 9-day-old seedlings were extracted with an RNA Prep Pure Plant kit (Tiangen, Shanghai, China). For reverse transcription, 1 mg of total RNA with oligo (dT) and random 6 mer primers were used to synthesize cDNAs in a 20 µL reaction using a M-MLV reverse transcriptase kit (TaKaRa, Shanghai, China). Quantitative PCR (qPCR) was performed using the SuperReal PreMix Color kit (Tiangen) in a CFX96 real-time system (Bio-Rad, CA, USA). All the primers used are included in Table S3.

## Figures and Tables

**Table 1:** Summary of number of differentially expressed proteins for each comparison.

Group Comparison	Regulated Type *	Fold Change >1.2	Fold Change >1.3	Fold Change >1.5	Fold Change >2
Col_TM/Col_CK	Upregulated	118	59	32	13
	Downregulated	72	29	4	1
L*er*_TM/L*er*_CK	Upregulated	135	82	44	14
	Downregulated	122	31	5	2
DM_TM/DM_CK	Upregulated	263	111	36	8
	Downregulated	130	57	11	0

*, Filtered with threshold value of fold change and *p* value (*p* < 0.05). TM, tunicamycin; CK, CHECK (control).

**Table 2:** Lists of proteins that specifically upregulated by ER stress in Col relative to L*er*.

Locus	Gene Name	Brief Description
AT1G27330	RAMP4	Ribosome associated membrane protein RAMP4
AT1G56340	CRT1A	One of three Arabidopsis calreticulins
AT1G65720	AT1G65720	Transmembrane protein
AT1G72280	ERO1	Oxidoreductin required for oxidative protein folding in the ER
AT2G02810	UTR1	Transporter of UDP-galactose and UDP-glucose into the Golgi
AT2G24980	EXT6	Proline-rich extensin-like family protein
AT2G29740	UGT71C2	UDP-glucosyl transferase 71C2
AT3G12250	TGA6	bZIP transcription factor involved in the activation of SA-responsive genes
AT3G16990	AT3G16990	Heme oxygenase-like protein
AT3G20920	SEC62	ER-localized protein with similarity to yeast Sec62p
AT3G51000	AT3G51000	Alpha/beta-hydrolases superfamily protein
AT4G29960	EBS7	Plant specific, ER-localized protein involved in ERAD
AT5G19250	AT5G19250	GPI-anchored glycoprotein
AT5G24590	NAC091	NAC family protein 091
AT5G25250	FLOT1	Protein involved in a membrane microdomain-dependent, but clathrin-independent, endocytic pathway
AT5G35080	OS9	Conserved ER-localized protein involved in ERAD
AT5G44120	CRA1	12S seed storage protein
AT5G55730	FLA1	Fasciclin-like arabinogalactan protein 1
AT5G64400	AT5G64400	CHCH domain protein involved in mechanotransduction

**Table 3:** Differential upregulation of proteins by ER stress in Col, L*er*, and DM plants.

Locus	Gene Name	Col_TM/Col_CK				L*er*_TM/L*er*_CK				DM_TM/DM_CK			
		Col_CK	Col_TM	Ratio	*p* Value	L*er*_CK	L*er*_TM	Ratio	*p* Value	DM_CK	DM_TM	Ratio	*p* Value
AT1G27330	At1g27330	0.66	1.34	2.02	0.0002	ND	ND			0.99	1.01	1.02	0.9587
AT1G56340	CRT1	0.86	1.16	1.35	0.0209	0.88	1.06	1.27	0.0070	0.98	1.02	1.05	0.2471
AT1G65720	At1g65720	0.85	1.15	1.35	0.0030	0.89	1.06	1.26	0.0428	0.88	1.10	1.25	0.0294
AT1G72280	ERO1	0.73	1.27	1.74	0.0004	0.96	1.02	1.09	0.4304	ND	ND		
AT2G02810	UTR1	0.63	1.37	2.18	0.0003	ND	ND			ND	ND		
AT2G24980	EXT6	0.82	1.18	1.44	0.0171	ND	ND			0.37	1.63	4.46	0.0001
AT2G29740	UGT71C2	0.86	1.14	1.32	0.0003	ND	ND			0.87	1.15	1.32	0.0027
AT3G12250	TGA6	0.75	1.25	1.65	0.0034	ND	ND			0.93	1.08	1.17	0.0239
AT3G16990	AT3G16990	0.88	1.15	1.31	0.0001	0.88	1.06	1.28	0.0096	1.05	0.95	0.90	0.1938
AT3G20920	SEC62	0.63	1.37	2.19	0.0003	ND	ND			ND	ND		
AT3G51000	AT3G51000	0.85	1.16	1.37	0.0249	1.00	1.00	1.00	0.9787	1.04	0.97	0.94	0.2280
AT4G29960	EBS7	0.84	1.16	1.39	0.0011	ND	ND			1.04	0.97	0.93	0.3135
AT5G19250	At5g19250	0.71	1.29	1.82	0.0028	1.02	0.99	0.95	0.4926	ND	ND		
AT5G24590	NAC091	0.85	1.15	1.36	0.0055	ND	ND			ND	ND		
AT5G25250	FLOT1	0.85	1.13	1.32	0.0076	0.87	1.06	1.29	0.0261	0.74	1.23	1.65	0.0008
AT5G35080	OS9	0.82	1.18	1.44	0.0463	ND	ND			0.98	1.02	1.04	0.4744
AT5G44120	CRA1	0.72	1.28	1.79	0.0203	ND	ND			1.00	1.00	1.00	0.9541
AT5G55730	FLA1	0.85	1.15	1.35	0.0170	0.89	1.05	1.25	0.1114	0.94	1.06	1.13	0.0085
AT5G64400	AT5G64400	0.83	1.17	1.41	0.0006	0.86	1.03	1.26	0.0398	0.93	1.07	1.14	0.2575

ND, not detected. Parameters were set as fold change >1.3 (upregulation) or <0.7 (downregulation), *p* < 0.05.

**Table 4:** Lists of proteins that specifically downregulated by ER stress in Col relative to L*er*.

Locus	Gene Name	Brief Description
AT1G11130	STRUBBELIG	Atypical receptor-like kinase protein
AT1G22200	AT1G22200	ER vesicle transporter protein
AT1G28600	AT1G28600	GDSL-motif esterase/acyltransferase/lipase
AT1G66270	BGLU21	Beta-glucosidase that has a high level of activity against the naturally occurring secondary metabolite scopolin
AT1G66280	BGLU22	Glycosyl hydrolase superfamily protein
AT1G76540	CDKB2;1	Cyclin-dependent protein kinase
AT2G04570	OSP1	GDSL-motif esterase/acyltransferase/lipase
AT2G15050	LTP7	PR (pathogenesis-related) protein
AT2G17790	VPS35A	Protein with similarity to yeast VPS35 involved in retrograde endosomal transport
AT2G19170	SLP3	Subtilisin-like serine protease
AT2G31360	ATADS2	Protein homolog of delta 9 acyl-lipid desaturase in cyanobacteria and acyl-CoA desaturase in yeast and mammals
AT3G17790	PAP17	Purple acid phosphatase 17
AT3G20560	PDIL5-3	Protein disulfide isomerase-like (PDIL) protein
AT3G61990	OMTF3	Protein methyltransferase
AT4G11650	OSM34	Osmotin-like protein
AT4G21960	PRXR1	Secretory peroxidase
AT4G23820	AT4G23820	Pectin lyase-like superfamily protein
AT4G24670	TAR2	Protein with similarity to the TAA1 trytophan aminotransferase involved in IAA biosynthesis
AT5G11460	FLZ10	FCS like zinc finger 10 induced during energy starvation through SnRK1 signaling
AT5G13930	CHS	Chalcone synthase (CHS) involved in the biosynthesis of flavonoids
AT5G14920	GASA14	GASA domain protein that regulates plant growth through GA-induced and DELLA-dependent signal transduction
AT5G24780	ATVSP1	Acid phosphatase
AT5G25610	RD22	Responsive to dehydration 22 (RD22) mediated by ABA
AT5G64620	VIF2	Cell wall/vacuolar inhibitor of fructosidase 2

**Table 5:** Differential downregulation of proteins by ER stress in Col, L*er,* and DM plants.

Locus	Gene Name	Col_TM/Col_CK				L*er*_TM/L*er*_CK				DM_TM/DM_CK			
		Col_CK	Col_TM	Ratio	*p* Value	L*er*_CK	L*er*_TM	Ratio	*p* Value	DM_CK	DM_TM	Ratio	*p* Value
AT1G11130	STRUBBELIG	1.14	0.86	0.75	0.0233	ND	ND			ND	ND		
AT1G22200	At1g22200	1.16	0.88	0.76	0.0020	1.12	0.88	0.79	0.0031	1.05	0.95	0.91	0.0311
AT1G28600	At1g28600	1.15	0.85	0.74	0.0001	1.06	0.94	0.89	0.0236	0.85	1.15	1.34	0.0053
AT1G66270	BGLU21	1.13	0.86	0.76	0.0015	1.11	0.89	0.80	0.0003	1.02	0.98	0.96	0.3381
AT1G66280	BGLU22	1.16	0.85	0.73	0.0000	1.11	0.89	0.80	0.0024	1.18	0.82	0.70	0.0002
AT1G76540	CDKB2;1	1.16	0.84	0.72	0.0011	1.09	0.91	0.84	0.0206	1.04	0.96	0.92	0.2542
AT2G04570	OSP1	1.15	0.85	0.74	0.0021	ND	ND			ND	ND		
AT2G15050	LTP7	1.13	0.87	0.77	0.0089	1.04	0.97	0.93	0.5423	0.94	1.07	1.14	0.0853
AT2G17790	VPS35A	1.15	0.85	0.75	0.0098	1.07	0.94	0.88	0.0403	0.96	1.04	1.08	0.4725
AT2G19170	SLP3	1.16	0.84	0.72	0.0053	ND	ND			0.97	0.98	1.01	0.9988
AT2G31360	ATADS2	1.21	0.79	0.65	0.0041	ND	ND			1.25	0.75	0.60	0.0145
AT3G17790	PAP17	1.14	0.86	0.76	0.0033	ND	ND			0.98	1.02	1.04	0.4345
AT3G20560	PDIL5-3	1.15	0.85	0.74	0.0263	ND	ND			0.98	1.01	1.03	0.1384
AT3G61990	OMTF3	1.42	0.58	0.41	0.0002	1.10	0.90	0.82	0.0399	0.95	1.03	1.08	0.4303
AT4G11650	OSM34	1.14	0.86	0.76	0.0073	1.10	0.90	0.81	0.0008	1.13	0.87	0.77	0.0282
AT4G21960	PRXR1	1.17	0.84	0.72	0.0001	1.14	0.89	0.78	0.0011	1.19	0.82	0.69	0.0015
AT4G23820	AT4G23820	1.13	0.86	0.76	0.0047	1.02	0.95	0.94	0.1996	1.00	0.98	0.98	0.4104
AT4G24670	TAR2	1.23	0.77	0.63	0.0034	1.10	0.90	0.81	0.0149	1.01	0.99	0.98	0.4490
AT5G11460	FLZ10	1.14	0.86	0.75	0.0020	1.04	0.97	0.93	0.1854	1.00	1.00	0.99	0.8603
AT5G13930	CHS	1.13	0.86	0.77	0.0220	1.09	0.89	0.82	0.0048	1.00	1.00	1.01	0.9969
AT5G14920	GASA14	1.14	0.86	0.76	0.0315	1.07	0.96	0.90	0.1817	0.94	1.06	1.14	0.0679
AT5G24780	ATVSP1	1.22	0.78	0.65	0.0003	ND	ND			0.97	1.03	1.06	0.2385
AT5G25610	RD22	1.16	0.84	0.73	0.0039	1.13	0.87	0.77	0.1557	1.01	1.00	0.99	0.7612
AT5G64620	VIF2	1.19	0.81	0.68	0.0007	1.13	0.87	0.77	0.0012	1.18	0.82	0.70	0.0002

ND, not detected. Parameters were set as fold change >1.3 (upregulation) or <0.7 (downregulation), *p* < 0.05.

## Data Availability

The raw data supporting the conclusions of this article will be made available by the authors, without undue reservation, to any qualified researcher.

## Supplementary Materials

The Supplementary Material for this article can be found online at: https://www.mdpi.com/1422-0067/21/24/9741/s1.
