# Supplementary material for: Quantitative Proteomic Analysis of ER Stress Response Reveals both Common and Specific Features in Two Contrasting Ecotypes of Arabidopsis thaliana
Source: Int J Mol Sci. 2020 Dec 21;21(24):9741. doi: 10.3390/ijms21249741 (PMC7766468; doi:10.3390/ijms21249741)
Supplement: Supplementary file 1 [file ijms-21-09741-s001.zip › IJMS SUPPL FIGURES.pdf]

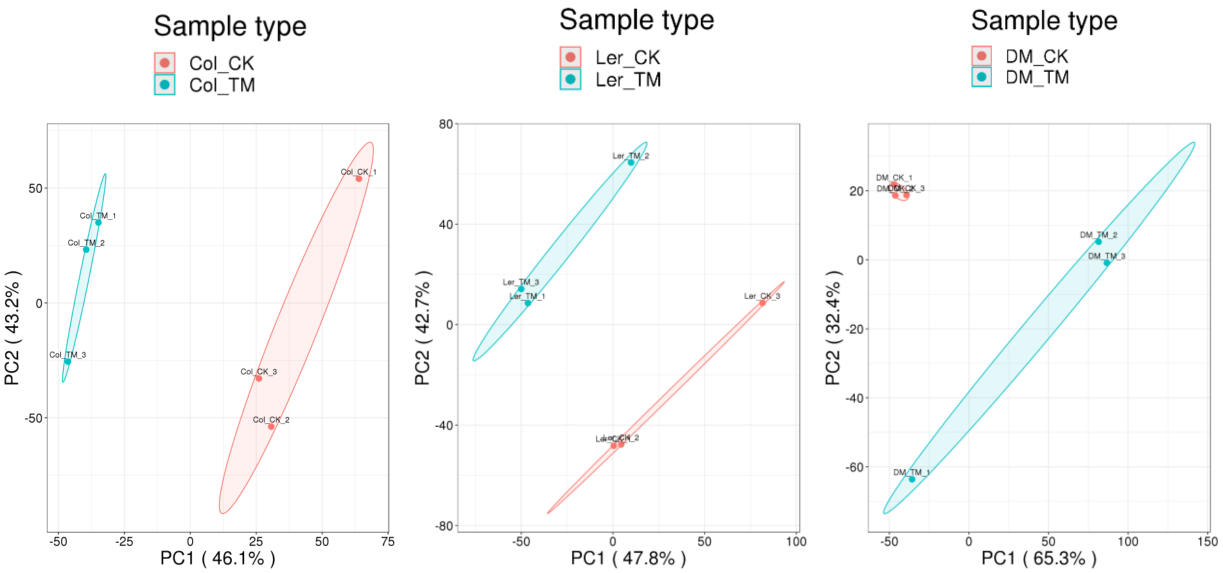

Supplementary Figure 1 | Principal components analysis (PCA) of samples from three replicates across three group of plants.

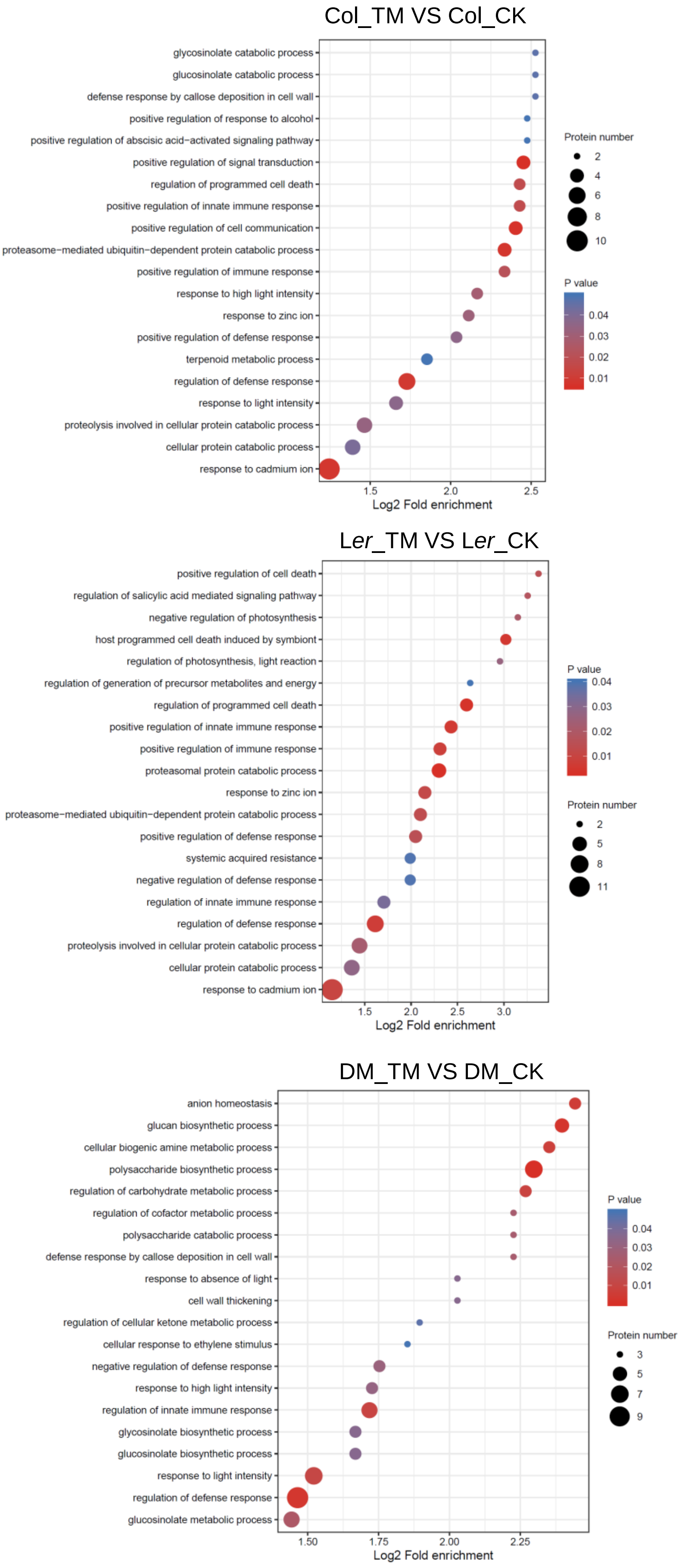

Supplementary Figure 2 | Functional enrichment (biological process) analysis of differentially expressed proteins between TM-treated and control (CK) plants.

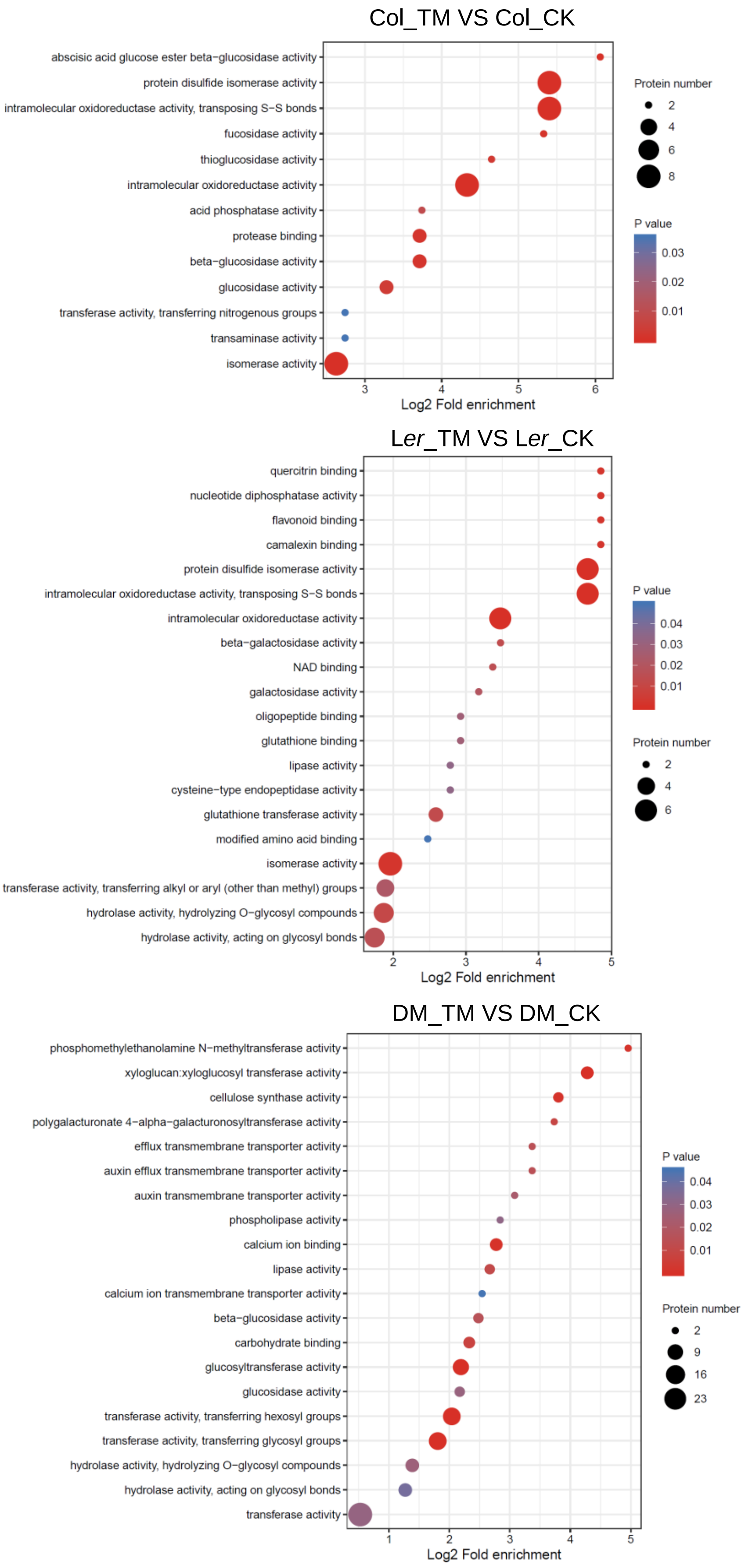

Supplementary Figure 3 | Functional enrichment (molecular function) analysis of differentially expressed proteins between TM-treated and control (CK) plants.

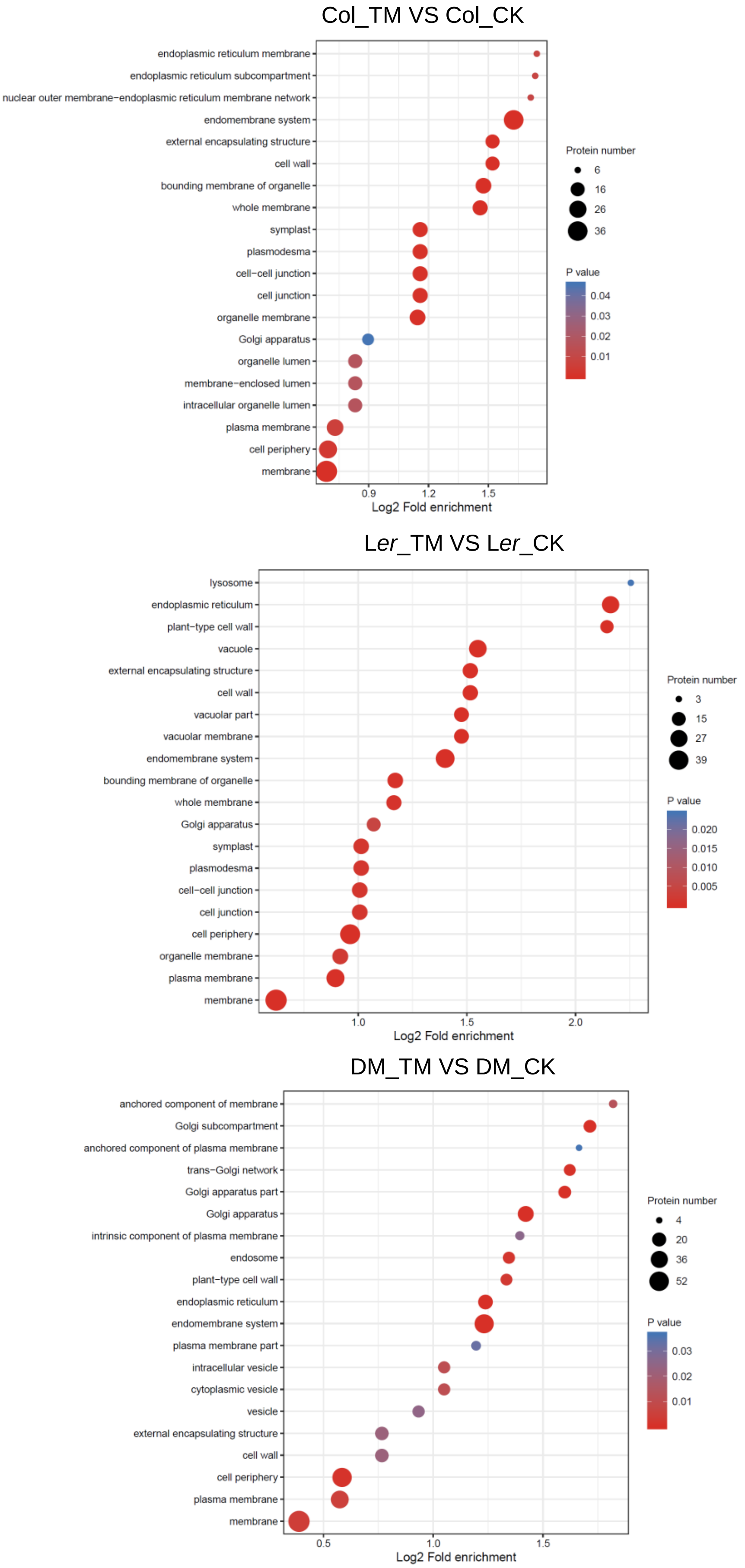

Supplementary Figure 4 | Functional enrichment (cellular component) analysis of differentially expressed proteins between TM-treated and control (CK) plants.
